# Preliminary and Graduating Requirements—the Logical Standard

**Published:** 1898-10

**Authors:** M. F. Ault

**Affiliations:** Dean of the Central College of Dentistry, Indianapolis


### Preliminary and Graduating Requirements—The Logical Standard.

BY M. F. AULT, INDIANAPOLIS.

Dean of the Central College of Dentistry.

Attention is first called to the principle, that in this country each one is entitled to the pursuit of happiness, and that the rules of an organization are not in harmony with the principles of life in the United States if they defeat the laudable aspirations of a young man or render void the law of natural selection.

There seems to be quite a tendency on the part of those whose duty it is to prescribe the terms of good repute among dental colleges, to move along the line of honest endeavor, but when the plan proposed is compared with our principles of life and common educational advancement in the different states it is certainly found to be excessive and arbitrary.

It is proposed by the National Examiners to increase the preliminery requisements until they shall be equivalent to high school graduation.

I am sure that while this is honestly entertained as a necessary step that it is beyond the advantages offered by the people in their means of public education, and that this is not only true in those states without high schools, but in most sections of the North the high school privilege is a luxury.

A high school diploma will not make a better student or more efficient dentist than an honest rank won in the common English branches, and which, so far as I know, every township in the country offers her children free.

Suppose a young man from a township provided with good common school advantage, and who has passed with credit those requirements, should decide to study dentistry. Is there a just reason for excluding him? To me there is as good reason for exacting a university degree as a high school diploma.

The plan of high school requirements will not only defeat many desirable students, but if honestly enforced by any college management it will defeat the school, and I believe that it will not only prove disastrous to new schools, but those with age and large attendance, if they rigorously enforce it, will be ruined. Why does this seem so? Because the requirement is beyond the public means of general education, and no school can select a sufficient number of students from high school graduates to enable it to survive.

Of course, dental departments of state universities can do that which a private institution could not, with our present competition and hazard in business, attempt.

Ignorant students are undesirable, and we can depend upon the natural instincts of an organization to protect itself by casting off ignorance which is so dense as to prove an obstruction to the work of the school. But this is not a plea to admit *any* who might apply, but to place the standard of admission where it will be equal to the means offered in our common school system, and to place the standard of graduation where it will be equal to the demands of a highly skilled and intelligent profession.

Any person who can pass a good examination in the eight common English branches can secure a license to teach, and it seems to me that a test in those branches which are required of persons engaged in the public service ought to be sufficient intellectual equipment to study those branches which belong to the dental course.

This becomes more apparent in view of the small number of males that graduate in high school. A class of fifty will remain intact until they finish the common course or the A grammar grade, and during the four years of high school the number and the ratio between male and female is so reduced that the graduating class will probably have four boys and sixteen girls. The unusually large class graduated from the Indianapolis High School in 1898 number one hundred and twelve; and of these there were only twenty-two young men. So I believe that the *logical maximum requirements* for admission to dental colleges is to be found in compliance with the general school law.

I wish to suggest a plan for admission and graduation which will, if adopted, not only possess the merit of being simple and inexpensive but will give great impetus to uniform and substantial progress.

(a) Make no excessive entrance requirements but decide upon a standard which will be so satisfactory to all recognized schools that none will resort to slippery tactics as a means of self-defence, and that will give all worthy persons a chance to prepare themselves for examination before the National Examiners. (I mean that a dental college should be a place of preparation only, and that in no sense should a school be the agent to determine fitness for practice.)

(b) Let the National Examiners prepare the graduation questions, these questions to be the same for all colleges, and sent sealed to the Dean of each school. (Schools of the Association are apt to be made of same length of term and final examination could be set for the same date, except universities, and they can be provided with extra set of questions.)

(c) Let the National Examiners appoint some disinterested person to be known as Examination Inspector, to go with the Dean of the college and on morning of examination the national seal is to be broken, questions unfolded, examination conducted in the presence of the inspector.

(d) Let the graduation fee, which is often foolishly expended for opera house and worthless display, be applied to pay the examining committee and inspectors for their services.

(e) After the examination the inspector is to take charge of the papers, forward them to the National Examiners' headquarters, where they will be examined and graded and diploma issued in obedience to the supreme decision.

(f) Let each school be assessed in proportion to its number of candidates for the degree.

This plan is designed to give all an opportunity to study dentistry without unjust restriction, and to place students and teachers under the necessity of submitting to a disinterested and unsympathetic body. It will make better work on the part of students and enforce closer attention on the part of members or a faculty to know that the work of teacher and pupil is to be graded by men whose relation is so remote as to make all feel that merit is the only winning element.

Now, it is evident that we have two plans before us, one is local and the other national. The first allows each college to determine its own standard, while the other is a single national standard for all. The first guarantees the irregularity and confusion that may be expected from as many standards as there are colleges, and the other will guarantee uniformity in work and equality in recognition and respect, since the diplomas of the associated colleges will possess the value of national endorsement.

